# Factors influencing diagnosis and treatment of osteoporosis after a fragility fracture among postmenopausal women in Asian countries: a retrospective study

**DOI:** 10.1186/1472-6874-13-7

**Published:** 2013-02-14

**Authors:** Annie W Kung, Tao Fan, Ling Xu, Wei B Xia, Il Hyung Park, Hak Sun Kim, Siew Pheng Chan, Joon Kiong Lee, Leonard Koh, Yung Kuei Soong, Suppasin Soontrapa, Thawee Songpatanasilp, Thana Turajane, Marc Yates, Shuvayu Sen

**Affiliations:** 1 Department of Medicine|, The University of Hong Kong, Hong Kong, China; 2 Global Outcomes Research, Merck & Co., Inc, Whitehouse Station, USA; 3 Department of Obstetrics and Gynecology, Beijing Union Medical Hospital, Beijing, China; 4 Department of Endocrinology, Peking Union Medical College Hospital, Beijing, China; 5 Department of Orthopaedic Surgery, Kyung Pook National University Hospital, Daegu, Korea; 6 Department of Orthopaedic Surgery, Yongdong Severance Hospital, Seoul, Korea; 7 University Malaya Medical Center, Kuala Lumpur, Malaysia; 8 Assunta Hospital, Petaling Jaya, Malaysia; 9 Gleneagles Medical Centre, Singapore, Singapore; 10 Medical college, Chang Gung University, Kwei-Shan, Taiwan; 11 Orthopaedics and Rehabilitation medicine, Khon Kaen University, Khon Kaen, Thailand; 12 Phramongkutklao Army Hospital and College of Medicine, Bangkok, Thailand; 13 Department of Orthopedics, Faculty of Medicine, Ramathibidi Hospital, Mahidol University, Maharaj Nakorn Chiangmai Hospital & Chiang Mai University, Chiang Mai, Thailand; 14 The Research Partnership, Singapore, Singapore; 15 Global Human Health, Outcomes Research, WS2E-76, Merck Sharp and Dohme, One Merck Drive, 08889, Whitehouse Station, NJ, USA

**Keywords:** Osteoporosis, Bone density, Female, Fractures, Asia

## Abstract

**Background:**

A vast amount of literature describes the incidence of fracture as a risk for recurrent osteoporotic fractures in western and Asian countries. Osteoporosis evaluation and treatment after a low-trauma fracture, however, has not been well characterized in postmenopausal women in Asia. The purpose of this study was to characterize patient and health system characteristics associated with the diagnosis and management of osteoporosis among postmenopausal women hospitalized with a fragility fracture in Asia.

**Methods:**

Patient surveys and medical charts of postmenopausal women (N=1,122) discharged after a fragility hip fracture from treatment centers in mainland China, Hong Kong, Singapore, South Korea, Malaysia, Taiwan, and Thailand between July 1, 2006 and June 30, 2007 were reviewed for bone mineral density (BMD) measurement, osteoporosis diagnosis, and osteoporosis treatment.

**Results:**

The mean (SD) age was 72.9 (11.5) years. A BMD measurement was reported by 28.2% of patients, 51.5% were informed that they had osteoporosis, and 33.0% received prescription medications for osteoporosis in the 6 months after discharge. Using multivariate logistic regression analyses, prior history of fracture decreased the odds of a BMD measurement (OR 0.63, 95% CI 0.45-0.88). Having a BMD measurement increased the odds of osteoporosis diagnosis (OR 10.1, 95% CI 6.36-16.0), as did having health insurance (OR 4.95, 95% CI 1.51-16.21 for private insurance with partial self-payment relative to 100% self-payment). A history of fracture was not independently associated with an osteoporosis diagnosis (OR 0.80, 95% CI 0.56-1.15). Younger age reduced the odds of receiving medication for osteoporosis (OR 0.59, 95% CI 0.36-0.96 relative to age ≥65), while having a BMD measurement increased the odds (OR 1.79, 95% CI 1.23-2.61).

**Conclusions:**

Osteoporosis diagnosis and treatment in Asian countries were driven by BMD measurement but not by fracture history. Future efforts should emphasize education of general practitioners and patients about the importance of fracture.

## Background

The prevalence of osteoporosis increases markedly after age 50 in postmenopausal women in Asia [1-3]. In Korea the prevalence of osteoporosis measured by bone mineral density (BMD) scanning of the lumbar spine has been reported to be 31% in postmenopausal women 45–64 years, 53% in those 65–74 years, and 69% in those ≥75 years [3]. Similar observations have been made in Taiwan [1].

A definitive diagnosis of osteoporosis in asymptomatic women is achieved via BMD measurement but, because of the relatively high cost of these measurements, a case-finding strategy is recommended [4]. A simple risk index based only on age and body weight can be used to screen for osteoporosis in Asian postmenopausal women prior to BMD measurement [5-8]. Since prior fracture is a well-documented risk factor for a future non-vertebral osteoporotic fracture in postmenopausal women in western and Asian countries [9-11], therefore, it is recommended that after a fragility fracture all postmenopausal women should be evaluated for osteoporosis [12]. Recent studies in western countries indicate that rates of treatment with medications for osteoporosis after a fracture vary widely. In a study in Italy 78% of patients received a medication for osteoporosis after a hip fracture [13]. Conversely, in the Netherlands only 19% of women age ≥50 years were treated with medications for osteoporosis in the year after a low-trauma fracture [14] and in Belgium only 6% of postmenopausal women received a bisphosphonate or hormone therapy after a hip fracture [15].

Little is known, however, about osteoporosis evaluation and treatment after a low-trauma fracture in countries in Asia. The objective of this study was to evaluate the diagnosis and treatment of osteoporosis among postmenopausal women after hospitalization for a fragility fracture in a selection of Asian countries.

## Methods

### Study design and patient sample

This was a patient survey and medical chart review carried out at multiple centers in six Asian countries including mainland China, Hong Kong, Singapore, South Korea, Malaysia, Taiwan, and Thailand. Women who were hospitalized due to fragility fracture of the hip between July 1st, 2006 and June 30th, 2007 (the case identification period) were identified using discharge records of participating hospitals. A random sample of these patients was selected for inclusion in the study. Patients were included if they had been hospitalized because of non-traumatic hip fracture (defined as resulting from a fall from standing height), were postmenopausal, and were able to participate independently in an interview. The index fracture, i.e., the fracture qualifying the subject for enrollment, was identified by reviewing hospitalization, emergency room or outpatient visit records for ICD-9/10 diagnosis codes 820.0, 820.2, or 820.8. Approval was obtained from participating hospital IRB committees; informed consent was provided by all patients.

### Data collection

#### Medical chart review

Medical charts were reviewed during hospitalization to obtain data on BMD (T-score for spine, femoral neck, or total hip), height and weight, comorbidities, and medications prescribed for osteoporosis.

#### Patient questionnaire

Face-to-face and telephone interviews were conducted with patients to collect data about an osteoporosis diagnosis, BMD measurement, and osteoporosis treatment in the 6-month period following hospitalization for fragility fracture. Patients were asked “Have you ever been told that you have osteoporosis?” If they answered “yes”, they were asked when they were told they had osteoporosis (in relation to the index fracture). They were also asked whether and when they had a BMD measurement for osteoporosis and what reimbursement, if any, they received for their hospital stay. Response options were either items in a checklist or “yes”, “no”, or “don’t know”. Questions about their post-fracture follow-up included whether they were advised to take prescription drugs or dietary supplements in the 6 month period after hospitalization for the index fracture, whether they were still being treated by a physician for osteoporosis, and the specialty of the physician. Other items recorded their demographic and, clinical characteristics and their fracture history prior to the index fracture.

### Statistical analysis

The main outcome variables were binary variables (yes/no) for a BMD measurement, an osteoporosis diagnosis, and receipt of an osteoporosis medication. The associations between patient characteristics (age category, fracture history, and payment type) and the outcome variables were examined in univariate analyses; P values were computed using Fisher’s exact test. Logistic regression analyses were applied to identify significant factors independently associated with the outcome variables BMD measurement, osteoporosis diagnosis and receipt of medications. For the model with BMD screening as outcome variable, independent variables were age category, history of fracture, and payment type. Only patients who answered “yes” or “no” were included in the model with a BMD measurement as dependent variable; patients who responded “don’t know” were excluded. For the model with osteoporosis diagnosis as outcome variable, independent variables included were age category, fracture history, BMD measurement and type of payment. For the model with receipt of osteoporosis medications as outcome variable, independent variables included were age category, fracture history, fracture diagnosis timing, BMD measurement and type of payment.

A subgroup analysis assessed the impact of lower BMD on osteoporosis diagnosis and receipt of osteoporosis medications among patients whose BMD values were recorded. BMD values were expressed as a categorical variable, and Fisher’s exact test was applied. BMD of the total spine, femoral neck, or total hip was used in the stated order as available. A logistic regression model was applied to this patient subset, with osteoporosis treatment as dependent variable and the following independent variables: age category, a history of fracture, and T score category. Percent of patients using osteoporosis medications were calculated.

## Results and discussion

### Patient sample

#### Selection of sample

A total of 1,148 post-menopausal women hospitalized due to fragility fractures met the inclusion criteria and were enrolled in the study. Of these, 26 (2.3%) patients did not answer the question about an osteoporosis diagnosis and were excluded from the analysis. The data analysis was applied to the remaining 1,122 subjects.

#### Patient characteristics

Patient characteristics are presented in Table 1. The mean (SD) age was 72.9 (11.5) years. A history of fragility fracture prior to the hip fracture was recorded in 16.1% of patients, 52.9% had hypertension and 29.3% had diabetes. The most frequent types of payment were “partial self pay with social insurance” (40.8%) and “100% self pay” (23.2%).

**Table 1:** Patient characteristics

	**n**	**N**^**a**^	**(%)**^**b**^
Age category (years)			
<50	41	1,122	(3.7)
50-64	188	1,122	(16.8)
≥65	893	1,122	(79.5)
BMD measurement	316	837	(28.2)
Been told had osteoporosis	579	1,122	(51.5)
Prescription drug for osteoporosis	370	1,122	(33.0)
Histories			
History of fragility fracture	174	1,081	(16.1)
Falls in past 12 months	278	1,051	(24.8)
Family history of osteoporosis	70	777	(6.2)
Family history of fracture	113	872	(10.1)
Corticosteroid use	41	972	(3.7)
Comorbidities			
Arthritis	103	889	(9.2)
Hypertension	593	1,048	(52.9)
Diabetes	329	975	(29.3)
Stroke	125	920	(11.1)
Depression	36	894	(3.2)
Parkinson’s disease	32	892	(2.9)
Type of payment			
100% self pay	260	1,069	(23.2)
Partial self pay with social insurance	458	1,069	(40.8)
Partial self pay with private insurance	25	1,069	(2.2)
100% paid by social insurance	100	1,069	(8.9)
100% paid by private insurance	23	1,069	(2.0)
Hospital treatment free of charge	41	1,069	(3.7)
Others	162	1,069	(14.4)

^a^Number of “yes” or “no” responses. Patients who did not respond to questions were treated as missing values and not included.

^b^As a percent of 1,122.

### BMD measurement

A BMD measurement prior to hip fracture was reported by 28.2% of the 837 patients who answered “yes” or “no” to the BMD measurement question (Table 1). Age category, a history of fracture, and type of payment were associated with having had a BMD measurement in the univariate analysis (Table 2). In the multivariate regression model (Table 3), the variables independently associated with having had a BMD measurement were fracture history (a history of fracture reduced the odds of a BMD measurement) and type of insurance (compared to 100% self pay, all specified types of insurance increased the odds of having a BMD measurement), but not age category.

**Table 2:** Rates of BMD measurement, osteoporosis diagnosis, and osteoporosis medication

	**BMD measurement (N=1,122)**		**Osteoporosis diagnosis (N=1,122)**		**Osteoporosis medication (N=1,122)**	
	**%**	**P value**^**a**^	**%**	**P value**^**a**^	**%**	**P value**^**a**^
Total	28.2		51.5		33.0	
Age		<0.001		0.009		
<50	9.8		17.1		0.0	
50-64	30.3		51.6		22.3	
≥65	28.7		53.1		35.5	
Fracture history		<0.001		<0.001		0.53
Yes (n=174)	38.5		65.5		36.8	
No (n=907)	26.9		48.6		32.0	
Payment type		<0.001		<0.001		<0.001
100% self pay	19.7		10.9		23.2	
Partial self pay with social insurance	25.9		21.2		40.8	
Partial self pay with private insurance	1.4		1.4		2.2	
100% paid by social insurance	7.3		3.3		8.9	
100% paid by private insurance	1.3		1.0		2.0	
Hospital treatment free of charge	3.4		2.5		3.7	
Others	11.2		9.6		14.4	
Unknown insurance	29.7		1.6		4.7	

^a^P-value for Fisher’s exact test.

**Table 3:** Logistic regression analyses

	**BMD measurement (N=837)**		**Osteoporosis diagnosis**		**Osteoporosis medication**	
	**OR**	**(95% CI)**	**OR**	**(95% CI)**	**OR**	**(95% CI)**
Age						
20- 49	1.38	(0.35 -5.51)	0.6	(0.18-2.00)	N/A	
50-64	1.36	(0.91-2.05)	1.19	(0.77-1.84)	0.59	(0.36-0.96)
65+	Reference		Reference		Reference	
Fracture history						
Yes	0.63	(0.45-0.88)	0.80	(0.56-1.15)	1.05	(0.71-1.54)
No	Reference		Reference		Reference	
Fracture diagnosis timing						
Before index fracture	N/A		N/A		0.99	(0.64-1.52)
During index fracture	N/A		N/A		1.38	(0.87-2.18)
After index fracture	N/A		N/A		Reference	
Unknown	N/A		N/A		0.28	(0.05-1.47)
A BMD measurement						
Yes	N/A		10.1	(6.36-16.0)	1.79	(1.23-2.61)
Unknown	N/A		0.06	(0.03-0.10	1.48	(0.59-3.74)
No	N/A		Reference		Reference	
Type of payment						
100% private insurance	8.19	(2.63 -25.58)	0.72	(0.20-2.54)	2.03	(0.54-7.66)
100% social insurance	1.96	(1.09 -3.50)	0.47	(0.27-0.84)	0.18	(0.07-0.51)
Other type of insurance	5.08	(3.11 -8.30)	2.48	(1.45-4.25)	2.45	(1.40-4.29)
Unknown insurance	1.33	(0.62-2.83)	0.39	(0.19-0.80)	1.05	(0.38-2.92)
Free hospital, government pay	4.13	(2.00-8.54)	1.31	(0.57-3.03)	1.56	(0.66-3.70)
Partial self with private insurance	3.43	(1.19-9.86)	4.95	(1.51-16.21)	0.49	(0.15-1.59)
Partial self with social insurance	3.94	(2.61 -5.95)	1.89	(1.28-2.81)	0.72	(0.45-1.15)
100% self pay	Reference		Reference		Reference	

N/A, not applicable.

### Osteoporosis diagnosis

Over half of the patients (51.5%) reported that they had been told they had osteoporosis (Table 1). Older age category, a history of fracture, and type of payment were significantly associated with an osteoporosis diagnosis (Table 2). In logistic regression analysis having had a BMD measurement and type of payment were associated with an osteoporosis diagnosis: compared to individuals with 100% self pay, the odds of an osteoporosis diagnosis were decreased among patients with 100% social insurance and increased among patients with partial self payment with private or social insurance, and among those with “other type of insurance” (Table 3). However, a fracture history was not significantly associated with an osteoporosis diagnosis. T scores were available for 256 patients. A lower T score was more likely associated with an osteoporosis diagnosis in the univariate analysis (Table 4).

**Table 4:** Osteoporosis diagnosis and treatment among the subgroup of patients with a BMD measurement

**T score**	**n (%)**	**Osteoporosis diagnosis (%)**^**a**^	**Osteoporosis medication (%)**^**a**^
Greater than −1	23 (9.0)	60.9	43.8
−1 to −2.5	103 (40.2)	70.0	59.2
−2.5 to −3	39 (15.2)	92.3	61.5
Lower than −3	91 (35.6)	82.4	78.0
Total	256 (100)	77.0	64.8

^a^P=0.003 (Fisher’s exact test).

### Osteoporosis treatment

Only 33.0% of patients received prescription medications for osteoporosis in the 6 months after the index fracture (Table 1). A bisphosphonate was prescribed for 26% of patients, representing 79% of prescription medications for osteoporosis (Table 5). Calcitonin was the most frequent non-bisphosphonate therapy, used by 4.8% of patients. Half of the patients (50.3%) used dietary supplements (Table 5).

**Table 5:** Osteoporosis treatments

	**(%) (N=1,122)**
Prescription medications for osteoporosis^a^	
Any prescription drugs	(33.0)
Any bisphosphonate	(26.0)
Any anti-resorptive	(31.0)
Alendronate	(16.5)
Risedronate	(6.9)
Calcitonin	(4.8)
Pamidronate	(4.2)
Raloxifene	(2.2)
Others	(2.9)
Supplement use	
Any supplement	(50.3)
Calcium	(32.1)
Vitamin D	(23.8)
Multi-vitamin	(9.1)

^a^Medications used by >2.0% of patients are listed.

Patient age category and payment type were significantly associated with treatment for osteoporosis in univariate analyses (Table 2). Age category, having had a BMD measurement, fracture history and type of payment were associated with osteoporosis treatment in multivariate logistic analysis (Table 3). Younger age category (50–64 years) reduced the odds of receiving medication for osteoporosis, while having had a BMD measurement increased the odds of treatment. The odds of treatment were reduced by having 100% social insurance and increased by “other type of insurance” when compared to 100% self pay. A history of fracture was not associated with receiving medications for osteoporosis. A more negative T score was associated with osteoporosis treatment in the univariate analysis (Table 4).

### Rates of BMD measurement, osteoporosis diagnosis and treatment, by country

**Figure 1:**
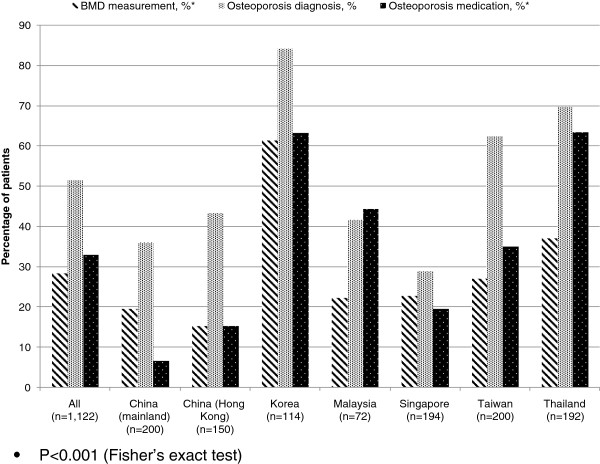
**BMD measurement, osteoporosis diagnosis and treatment, by country.** P<0.001 (Fisher’s exact test).

Korea and Thailand were the top ranking countries for rates of BMD measurement, osteoporosis diagnosis, and osteoporosis treatment (Figure 1). Malaysia was the only country in which the rate of osteoporosis treatment exceeded the rate of diagnosis. Rates of diagnosis and treatment exceeded rates of BMD testing in all countries except mainland China and Singapore.

In this population of elderly (average age 72.9 years) women discharged from hospital after a fragility fracture, having a BMD measurement (28.2% of patients) increased the odds of a diagnosis of osteoporosis and treatment. Compared to 100% self-payment, having any known type of health insurance increased the odds of a BMD measurement-suggesting an unwillingness to pay out of pocket for preventive care-while having 100% social insurance reduced both the odds of receiving prescription medicines for osteoporosis and the odds of an osteoporosis diagnosis-almost half (48.5%) of the patients had not been told that they had osteoporosis.

Among the 41% of patients who reported their physician’s specialty, the same percentage (29%) of orthopedic specialists and general practitioners (GPs) prescribed medications for osteoporosis. However, information about osteoporosis was communicated to patients largely by orthopedic specialists (76%) rather than by GPs (11%), suggesting that GPs need more education about osteoporosis.

Results of this study suggest that greater attention should be paid to patients with a history of fracture. A history of fracture, recorded for 13.2% of patients, was not significantly associated with an osteoporosis diagnosis independently of other factors-BMD, age, payment type-or with receiving medications for osteoporosis medications after the index fracture. The only statistically significant sequelae of a history of prior fracture was that it reduced the odds of a BMD measurement-perhaps because physicians deduced that a prior fracture was indicative of osteoporosis, obviating the need for a BMD test. A recurrent fracture after the index fracture was experienced by 9% of the patients in this study. A history of prior fracture was a significant independent predictor of a recurrent facture (OR 1.67, 95% CI 1.02-2.72), while other factors (age, an osteoporosis diagnosis, a BMD measurement) were not significantly predictive.

## Summary

In this study, an average of 33.0% of patients received a prescription medication for osteoporosis in the 6 months after the index fracture, though this varied from 6.5% in mainland China to 63.5% in Thailand. These rates are comparable or better than recent practice recorded in Western countries. In a 2003–04 UK study of patients (97% women) after a fracture of the distal radius, 8.5% were referred for BMD scanning, calcium and vitamin D was prescribed to 22%, and bisphosphonates to 9% [16]. In a 2006 study set in Sweden, management of patients post fracture depended on the results of a BMD scan [17]. In the three years after the fracture, antiresorptive medicines (excluding calcium-vitamin D) had been received by two thirds of osteoporotic, one sixth of osteopenic, and none of the patients with normal BMD [17]. There is little recent data for the United States, and most studies of medical practice after low-trauma or hip fracture relate to small single-center studies in the 1990s. Among these US studies the median rate of prescribed medicines for osteoporosis after a fracture was 24% (range 5-60%) [18-24].

## Conclusions

Even after experiencing a fragility fracture, 48.5% of Asian postmenopausal women reported that they had not been told that they had osteoporosis and only 33.0% were receiving prescription medications for osteoporosis. A history of fracture did not increase the odds of an osteoporosis diagnosis or treatment, indicating that both patients and GPs should be educated about the importance of fractures in the evaluation of osteoporosis.

## Abbreviations

AK: A.W. Kung; AM: A. Modi; BMD: Bone mineral density; GPs: General practitioners; HK: H.S. Kim; ICD: International Statistical Classification of Diseases and Related Health Problems; IP: I.H. Park; IRB: Institutional review board; JL: J.K. Lee; LK: L. Koh; LX: L. Xu; MY: M. Yates; SC: S.P. Chan; SS: S. Soontrapa; SSS: S. Sen; TF: T. Fan; TS: T. Songpatanasilp; TT: T. Turajane; UK: United Kingdom; WX: W.B. Xia; YS: Y.K. Soong.

## Competing interests

TF, AM, and, SSS are employees of Merck & Co., Inc. The other authors have no conflicts of interest to declare.

## Authors’ contributions

AK, TF, and LX contributed to study design, manuscript writing, patient recruitment, questionnaire development, and statistical analysis. WX, IP, HK, SC, JL, LK, YS, SS, TS, and TT contributed to study design, manuscript writing, patient recruitment, and questionnaire development. MY contributed to study design, manuscript writing, and questionnaire development. AM contributed to manuscript writing and SSS contributed to study design and questionnaire development. All of the authors reviewed and approved the final manuscript.

## Pre-publication history

The pre-publication history for this paper can be accessed here:

http://www.biomedcentral.com/1472-6874/13/7/prepub
